# Alternative Laser Treatment Protocol for Bruise Management

**DOI:** 10.1111/jocd.70299

**Published:** 2025-06-19

**Authors:** Kyu‐Ho Yi, Jovian Wan, Song Eun Yoon, Ruri Pamela, Hugues Cartier, Sebastien Garson, Benjamin Ascher

**Affiliations:** ^1^ Division in Anatomy and Developmental Biology, Department of Oral Biology, Human Identification Research Institute, BK21 FOUR Project Yonsei University College of Dentistry Seoul Korea; ^2^ You and I Clinic Seoul Korea; ^3^ Medical Research Inc. Wonju Korea; ^4^ BRANDNEW Aesthetic Surgery Clinic Seoul Korea; ^5^ CELV Dermatology Clinic Jakarta Indonesia; ^6^ Centre Médical Saint Jean Arras France; ^7^ Cabinet Médical Senlis France; ^8^ SibUS‐In Paris France

**Keywords:** bruises, cryotherapy, hematoma, laser therapy, low‐level, purpura

## Abstract

**Background:**

Post‐procedural ecchymosis can last up to two weeks and diminish patient satisfaction. Laser‐assisted clearance is documented with pulsed‐dye devices, but data on 1064‐nm picosecond/nanosecond platforms remain sparse.

**Objective:**

To evaluate a staged 1064‐nm laser protocol for accelerating bruise resolution after dermal filler injections.

**Methods:**

Three women (aged 40–52 years, Fitzpatrick III–IV) with facial bruises were treated on Day 2–3 using a picosecond 1064‐nm Nd:YAG laser (0.20–0.30 J/cm², 8–10 mm spot, 8–10 Hz, 8–10 min). A nanosecond Q‐switched 1064‐nm Nd:YAG session followed on Day 4–7 (2.5–3.0 J, 8 mm spot, 30–40 stacked shots) with peri‐session cooling. Clinical photographs and Global Aesthetic Improvement Scale (GAIS) ratings were obtained through Day 7.

**Results:**

All bruises faded substantially within 4–5 days; near‐complete resolution was achieved by Day 6–7. GAIS scores were “Much” or “Very Much Improved” in every case. No pain, post‐inflammatory hyperpigmentation, or other adverse effects occurred. Compared with typical spontaneous recovery (≈10–14 days), the protocol shortened visible downtime by 40%–60%.

**Conclusion:**

A sequential 1064‐nm picosecond–nanosecond regimen appears safe and effective for rapid ecchymosis clearance after aesthetic injections. Larger controlled studies are warranted to optimise fluence, cost‐effectiveness, and applicability to other procedural bruises and darker skin types.

## 1 Introduction

Bruising, or ecchymosis, frequently arises as a side effect of aesthetic procedures such as dermal filler injections and thread lifting. It results from the extravasation of blood into soft tissues and is characterized by visible discoloration ranging from reddish to bluish tones. While bruising typically resolves spontaneously within 10–14 days, it can persist longer in some cases, impacting patients' social confidence and satisfaction with the procedure [1, 2].

The management of post‐procedural bruising has traditionally relied on conservative methods, including the application of ice, topical agents such as vitamin K creams, or oral supplements such as arnica [3, 4]. These approaches, however, have shown limited efficacy in accelerating bruise resolution. Laser‐based treatments have emerged as a promising alternative, leveraging selective photothermolysis to target hemoglobin and disrupt extravasated blood. Pulsed‐dye lasers (PDL) have been widely reported for their effectiveness in bruise management, but the use of 1064 nm picosecond and nanosecond lasers is less documented [5, 6, 7]. This case report presents the successful use of a staged laser protocol to treat a bruise following nasolabial fold filler injection, demonstrating its potential as an effective and patient‐friendly solution.

### 1.1 Influence of Bruise Characteristics on Laser Response

The response to laser treatment for bruises is influenced by multiple factors, including the depth of the bruise, hematoma size, and the stage of resolution. Deeper hematomas may require different wavelengths or multiple treatment sessions compared to more superficial bruises. Early‐stage bruises rich in oxyhemoglobin respond differently than later‐stage bruises dominated by hemosiderin, necessitating adjustments in treatment parameters based on the bruise's evolution. The optical properties of the chromophore are also critical; the 1064 nm wavelength used in our staged protocol penetrates more deeply into the dermis and selectively targets hemoglobin degradation products while minimizing melanin absorption, making it suitable for a wider range of skin types. Additionally, the picosecond laser's ultra‐short pulse duration predominantly produces a mechanical effect rather than a thermal one, allowing for photomechanical disruption of hemosiderin with minimal risk of heat‐induced tissue injury. This feature is especially important for reducing the likelihood of post‐inflammatory hyperpigmentation in patients with darker Fitzpatrick skin types. Moreover, the use of a 10 mm spot size in the picosecond phase promotes deeper penetration and a more uniform energy distribution, optimizing treatment efficiency across varied bruise presentations. Given these considerations, individualized treatment protocols tailored to each patient's bruise characteristics—depth, hematoma volume, and resolution stage—are crucial for maximizing therapeutic outcomes and minimizing complications.

## 2 Case 1

A 52‐year‐old female patient presented with a bruise on the left side of her face following a cannula injection for nasolabial fold augmentation with hyaluronic acid filler. Despite the use of a blunt‐tipped cannula, subcutaneous bleeding occurred, leading to the development of a noticeable bruise. The patient expressed concerns about the aesthetic and social implications of the bruise and sought a treatment to expedite recovery. A progressive laser protocol was initiated, with sessions scheduled to align with the physiological evolution of the bruise.

### 2.1 Treatment Protocol

#### 2.1.1 Day 2–3: Initial Laser Intervention

- Device: 1064 ps laser (Picocare Majesty, Wontech, Korea)
- Rationale: Picosecond lasers utilize mechanical vibrations to target blood clots while minimizing thermal damage to surrounding tissues. This approach is particularly effective in the early stages of bruising.
- Parameters:
  ○ Fluence: 0.25 J/cm^2^
  ○ Spot size: 10 mm
  ○ Frequency: 10 Hz
  ○ Duration: 10 min
- Outcome: Initial lightening of the bruise was observed, with a reduction in redness and swelling by Day 3.

#### 2.1.2 Day 3–6: Follow‐Up Laser Session

- Device: QS Nd:YAG 1064 ns laser (Pastelle, Wontech, Korea)
- Rationale: Nanosecond lasers deliver focused energy to break down hemoglobin deposits and accelerate pigment clearance, targeting intermediate‐stage bruising.
- Parameters:
  ○ Energy: 3.0 J
  ○ Spot size: 8 mm
  ○ Treatment protocol:
    ■ Pre‐session ice application
    ■ 40 stacked shots
    ■ Post‐treatment cooling for 15 min
- Outcome: The bruise showed significant resolution, with most pigmentation cleared by Day 6, leaving minimal residual discoloration.
- GAIS Score: At Day 7, the patient and two independent evaluators rated the outcome as “Very Much Improved” on the Global Aesthetic Improvement Scale (GAIS).

## 3 Case 2

A 40‐year‐old female patient developed a moderate‐sized bruise on the right lower cheek following a cannula injection for midface volume restoration using hyaluronic acid filler. Despite careful technique with a blunt‐tipped cannula, subcutaneous vessel trauma resulted in a visible hematoma. The patient sought accelerated healing due to professional obligations. A staged laser protocol was applied, adapted to the clinical characteristics and evolution of her bruise.

### 3.1 Treatment Protocol

#### 3.1.1 Day 2–3: Initial Laser Intervention

**FIGURE 1:**
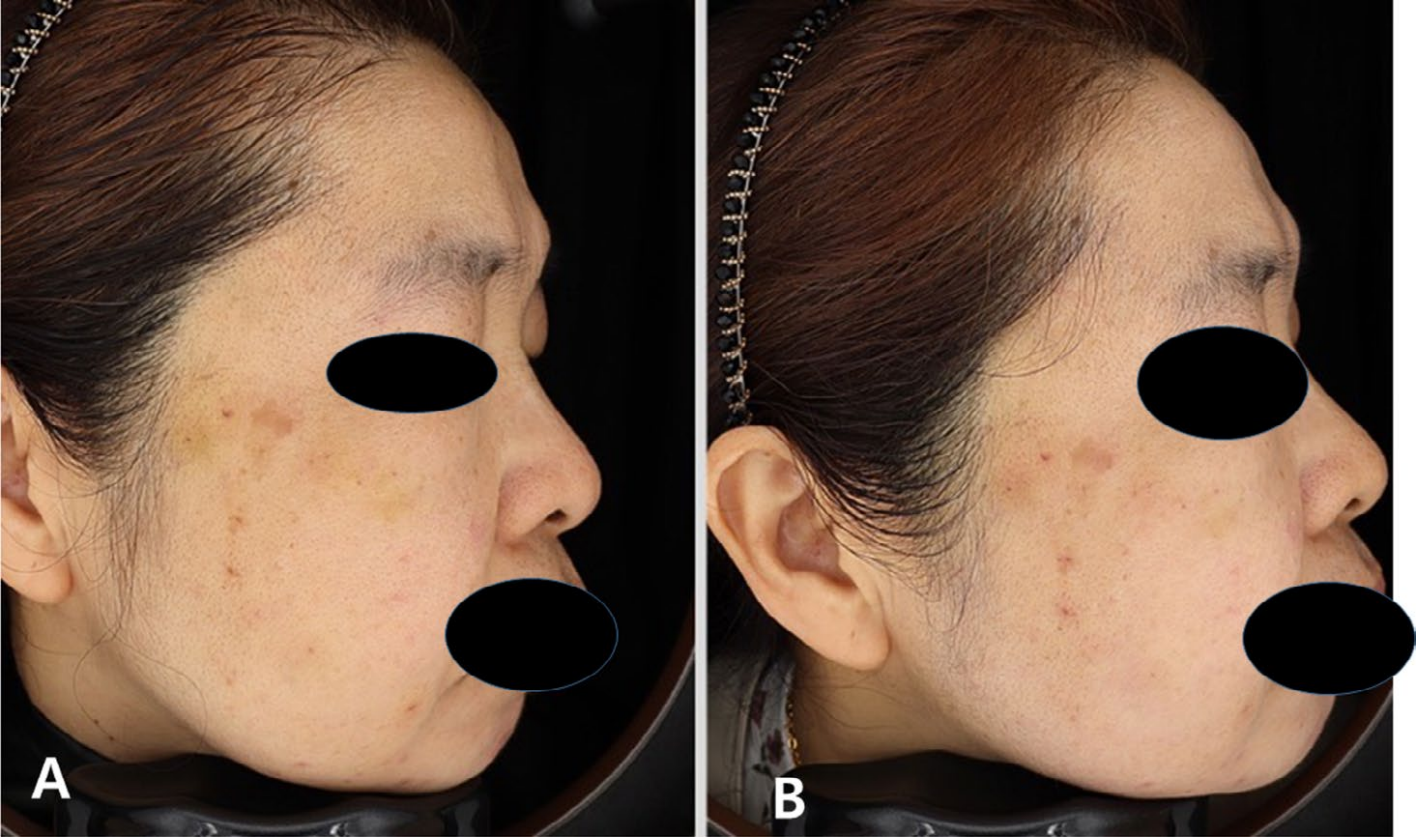
Immediate effect after picosecond laser treatment (PicoMajesty, Wontech Inc., Korea). Photographs taken immediately before (A) and immediately after (B) the treatment.

- Device: 1064 ps laser (Picocare Majesty, Wontech, Korea)
- Rationale: Mechanical photoacoustic effects of the picosecond pulse facilitate early‐stage clot disruption while limiting thermal injury, particularly effective in reducing the density of fresh bruising.
- Parameters:
  ○ Fluence: 0.30 J/cm^2^
  ○ Spot size: 10 mm
  ○ Frequency: 8 Hz
  ○ Duration: 8 min
- Outcome: Partial fading of the deep purple component of the bruise was noted by Day 3, with decreased swelling and improved skin tone (Figure 1).

#### 3.1.2 Day 4–7: Follow‐Up Laser Session

- Device: QS Nd:YAG 1064 ns laser (Pastelle, Wontech, Korea)
- Rationale: Nanosecond pulses were employed to further fragment residual hemosiderin and expedite clearance during the intermediate resolution phase.
- Parameters:
  ○ Energy: 2.5 J
  ○ Spot size: 8 mm
  ○ Treatment protocol:
    ■ Pre‐treatment cooling with ice packs
    ■ 35 stacked shots focused over the area
    ■ Post‐laser cooling for 10–15 min
- Outcome: By Day 7, the bruise had significantly diminished, with near‐complete resolution and only faint yellowish discoloration remaining, without any signs of post‐inflammatory hyperpigmentation.
- GAIS Score: At Day 7, the patient and two independent evaluators rated the outcome as “Very Much Improved” on the Global Aesthetic Improvement Scale (GAIS).

## 4 Case 3

A 45‐year‐old female patient developed a bruise on the left periorbital area following a tear trough filler injection with a blunt‐tipped cannula. Although care was taken during the procedure, minor vascular trauma led to a moderate‐sized hematoma, causing visible discoloration and swelling under the eye. The patient was concerned about prolonged downtime and sought accelerated recovery before an upcoming public event. A staged laser protocol was initiated, tailored to the anatomical location and clinical evolution of the bruise.

### 4.1 Treatment Protocol

#### 4.1.1 Day 2–3: Initial Laser Intervention

- Device: 1064 ps laser (Picocare Majesty, Wontech, Korea)
- Rationale: The mechanical disruption provided by the picosecond pulse was utilized to minimize early clot organization and facilitate early resolution without thermal damage, especially important for the delicate periorbital skin.
- Parameters:
  ○ Fluence: 0.20 J/cm^2^
  ○ Spot size: 8 mm (smaller spot size for precision in the periorbital area)
  ○ Frequency: 10 Hz
  ○ Duration: 8 min
- Outcome: By Day 3, noticeable lightening of the hematoma was achieved, with decreased swelling and partial fading of the bluish‐purple hue.

#### 4.1.2 Day 4–7: Follow‐Up Laser Session

- Device: QS Nd:YAG 1064 ns laser (Pastelle, Wontech, Korea)
- Rationale: Nanosecond pulses were used to further disrupt hemosiderin deposits and promote accelerated clearance in the intermediate healing phase.
- Parameters:
  ○ Energy: 2.8 J
  ○ Spot size: 6 mm (focusing more precisely in the periorbital region)
  ○ Treatment protocol:
    ■ Pre‐treatment cooling with ice packs
    ■ 30 stacked shots over the area
    ■ Post‐laser cooling for 10–15 min
- Outcome: By Day 7, the bruise had largely resolved, with only minimal residual yellowish discoloration that was easily concealed with light makeup. No adverse effects such as hyperpigmentation or hypopigmentation were observed.
- GAIS Score: At Day 7, the patient and two independent evaluators rated the outcome as “Much Improved” on the Global Aesthetic Improvement Scale (GAIS).

## 5 Results

**FIGURE 2:**
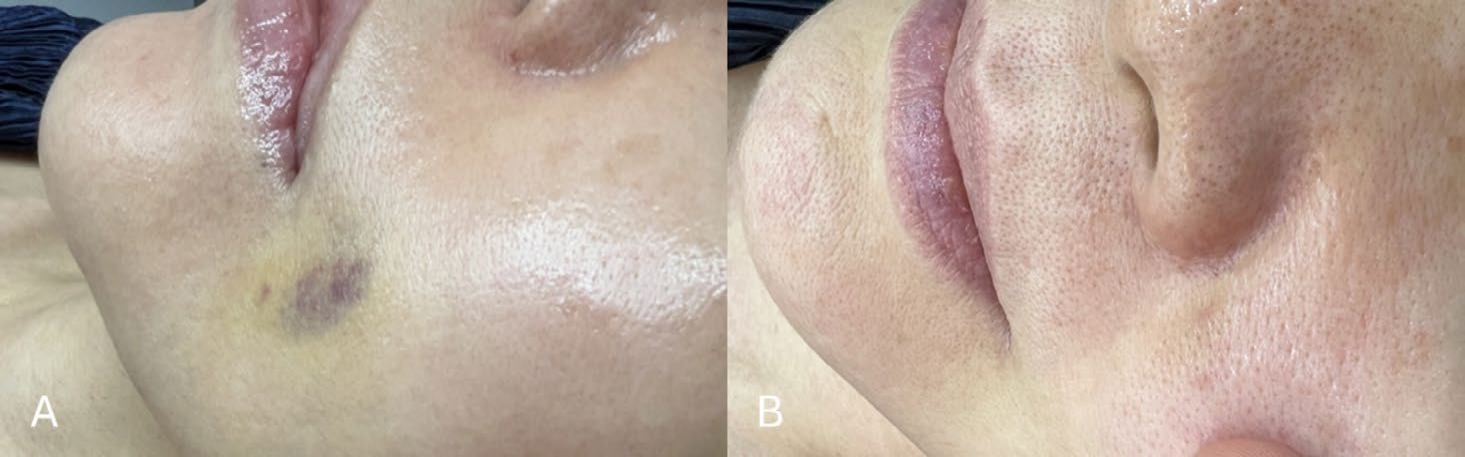
(A) Appearance of the bruise before the initial laser session, showing pronounced redness and discoloration. (B) Near‐complete resolution of the bruise by Day 6 following the staged laser treatment protocol.

Photographic documentation captured the progression of the bruise's resolution. Before and after images illustrate the effectiveness of the laser treatment (Figure 2). The patient reported no adverse effects, such as discomfort or hyperpigmentation, and expressed high satisfaction with the treatment outcome.

## 6 Discussion

This case highlights the efficacy of a staged laser treatment protocol specifically tailored to the evolutionary phases of bruising. Each laser modality was carefully selected to target distinct pathophysiological processes associated with the progression of ecchymosis. During the initial phase, spanning Days 2–3, the application of picosecond lasers proved effective in disrupting fresh blood clots through mechanical vibrations. This intervention facilitated the early breakdown of hemoglobin, minimizing tissue trauma and optimizing the outcomes of early‐phase treatment.

As the bruise transitioned into the intermediate phase, between Days 3 and 6, nanosecond lasers were employed to target residual hemoglobin deposits. By focusing on pigment clearance and reducing discoloration, this phase capitalized on the natural migration of hemoglobin from deeper to more superficial layers, enabling more efficient light absorption and pigment removal. The use of these modalities in a staged manner is aligned with the physiological progression of the bruise, providing a structured approach to its resolution [8].

Bruising is a common complication of aesthetic procedures, not limited to dermal fillers, but also frequently observed with thread lifting and botulinum toxin injections. Thread lifting, for example, often results in bruising at entry and exit points due to vascular injury during insertion. Similarly, botulinum toxin injections may cause ecchymosis when administered in highly vascularized areas or due to improper technique [1]. The laser‐based protocol demonstrated in this case could be extended to manage such bruises, broadening its applicability across various aesthetic interventions.

Compared to traditional approaches such as PDL or topical agents, the described protocol provided superior results with a significantly faster recovery timeline. Furthermore, the absence of adverse effects emphasizes its safety and suitability for routine aesthetic practice. The incorporation of cooling measures alongside laser therapy not only enhanced patient comfort but also mitigated the risk of post‐treatment inflammation, contributing to the overall effectiveness of the protocol.

While the findings from this case are promising, further research involving larger patient cohorts is necessary to refine and standardize the parameters for this treatment. Future studies should also investigate the cost‐effectiveness and accessibility of laser‐based bruise management to ensure broader clinical utility. Moreover, exploring the protocol's application for deeper or older bruises, as well as evaluating its outcomes across a diverse range of Fitzpatrick skin types, could further validate and expand its relevance in aesthetic medicine. This staged laser protocol thus represents a valuable advancement in managing post‐procedural bruising, with the potential to improve both clinical outcomes and patient satisfaction.

When applying laser treatments for bruising, particular caution is necessary in patients with darker skin tones (Fitzpatrick skin types IV–VI) due to the increased risk of post‐inflammatory hyperpigmentation (PIH). Melanin absorption, even with longer wavelengths like 1064 nm, remains a concern in these populations. To minimize this risk, lower fluence settings, larger spot sizes, and conservative treatment parameters should be used, especially during the early stages of healing when inflammation is highest. In addition, the use of picosecond lasers, which primarily produce mechanical effects rather than thermal injury, may offer a safety advantage by reducing the risk of pigmentary alterations. Careful patient selection, pre‐treatment counseling regarding potential pigmentary changes, and post‐treatment photoprotection are essential strategies to ensure safe and effective outcomes across diverse skin types.

### 6.1 Study Limitations and Cost Considerations

This report presents preliminary observations based on two patient cases without a control group or direct comparison to standard treatments such as PDL, limiting the generalizability of the findings. Although initial outcomes appear encouraging, larger studies with standardized photographic assessments, objective outcome measures, and direct comparisons with established therapies are needed to validate the efficacy and safety of this staged laser protocol. Additionally, the use of multiple devices and treatment sessions may increase the financial burden compared to traditional approaches or spontaneous resolution. While accelerated bruise clearance may offer important social and psychological benefits, the overall cost‐effectiveness of this approach remains to be evaluated through future studies incorporating formal economic analyses.

## 7 Conclusion

The progressive laser treatment protocol effectively managed post‐procedural bruising in this case, offering a rapid, safe, and patient‐friendly solution. By tailoring laser modalities to the stages of bruise evolution, this approach enhances recovery outcomes and patient satisfaction. This protocol holds promise as a valuable addition to aesthetic practice, with potential implications for broader clinical adoption.

## Author Contributions

Conceptualization: Kyu‐Ho Yi, Jovian Wan, Song Eun Yoon, Hugues Cartier, Sebastien Garson, Benjamin Ascher. Writing – Original Draft Preparation: Kyu‐Ho Yi, Jovian Wan. Writing – Review and Editing: Kyu‐Ho Yi, Jovian Wan, Song Eun Yoon, Hugues Cartier, Sebastien Garson, Benjamin Ascher. Visualization: Kyu‐Ho Yi, Jovian Wan, Song Eun Yoon, Hugues Cartier, Sebastien Garson, Benjamin Ascher. Supervision: Kyu‐Ho Yi. All authors have reviewed and approved the article for submission.

## Conflicts of Interest

The authors declare no conflicts of interest.

## Data Availability

The data that support the findings of this study are available on request from the corresponding author. The data are not publicly available due to privacy or ethical restrictions.
